# Supplementary material for: Afferent convergence to a shared population of interneuron AMPA receptors
Source: Nat Commun. 2023 May 30;14:3113. doi: 10.1038/s41467-023-38854-2 (PMC10229553; doi:10.1038/s41467-023-38854-2)
Supplement: Supplementary file 1 — Supplementary Information [file 41467_2023_38854_MOESM1_ESM.pdf]

Supplementary Information

Afferent convergence to a shared population of interneuron AMPA receptors

Reagan L. Pennock[1], Luke T. Coddington[1,2], Xiaohui Yan[1], Linda Overstreet-Wadiche[1*], and Jacques I. Wadiche[1*]

[1]Department of Neurobiology, University of Alabama at Birmingham,
Birmingham, Alabama 35294
[2]Howard Hughes Medical Institute Janelia Research Campus,
Ashburn, Virginia 20147

[*] corresponding authors:
Jacques I. Wadiche, Ph.D. or Linda Overstreet-Wadiche, Ph.D.
University of Alabama at Birmingham; Department of Neurobiology
Birmingham, AL 35294
email: jwadiche@uab.edu or lwadiche@uab.edu

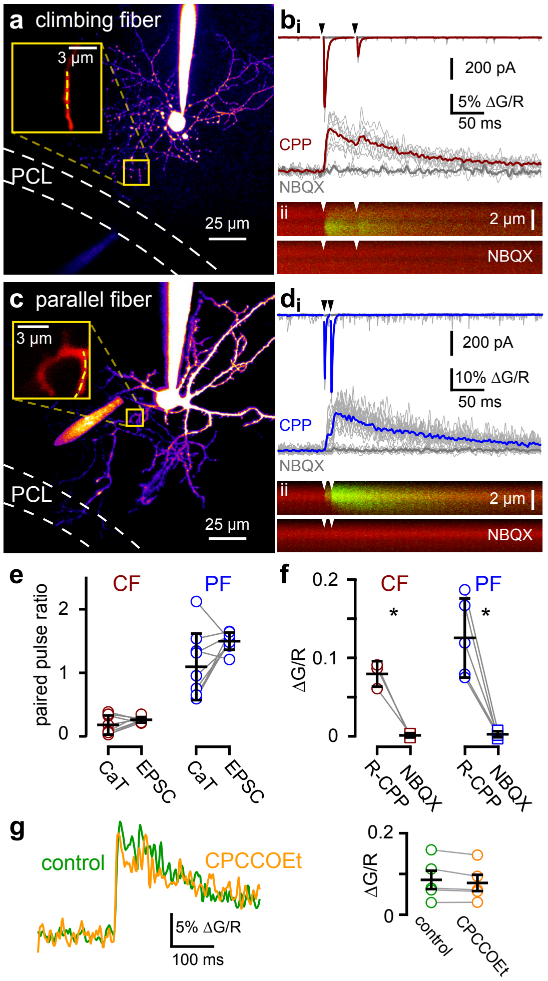

Supplementary Figure 1. CF- and PF-evoked CaTs mirror evoked EPSCs.

(a) 2P Z-projection of an MLI (inset: dendrite) receiving CF spillover. The dashed yellow line indicates the line scan path. **(b_i)** Individual (gray) and averaged (red) CF- EPSCs (top) and CaTs (bottom) in response to paired stimuli (inter-stimulus interval, ISI, 50 ms). Lines scans at 1 kHz. CF EPSCs and CaTs were blocked by NBQX (10 µM; dark gray traces). **(b_{ii})** Average line scan images corresponding the CaT shown in (b_i). Stimuli are indicated by arrowheads. **(c-d)** Similar images for PFs in response to paired stimuli (ISI 10 ms). **(e)** Summary of paired pulse ratio for CFs (red; PPR_{CaT} = 0.18 ± 0.053 vs. PPR_{EPSC} = 0.26 ± 0.015, n = 8, p = 0.20, Two-tailed paired t-test) and PFs (blue; PPR_{CaT} = 1.1 ± 0.18 vs. PPR_{EPSC} = 1.5 ± 0.045, n = 9, p = 0.06, Two-tailed paired t-test). **(f)** NBQX inhibition of CF- and PF-evoked CaTs (CF: 0.079 ± 0.0094 to 0.0011 ± 0.0015 ΔG/R, n = 3, p = 0.02, Two-tailed paired t-test; PFs: 0.13 ± 0.023 to 0.0025 ± 0.0017 ΔG/R, n = 5, p = 0.01, Two-tailed paired t test). Asterisk denotes $p < 0.05$. **(g)** Average CF CaTs before (green) and after CPCCOEt (orange). Summary CPCCOEt effect on CF CaT amplitude (0.09 ± 0.02 to 0.08 ± 0.02 ΔG/R, n = 5 CaTs from 3 cells, p = 0.09, Two-tailed paired t test). Data are shown as mean ± SEM. Source data are provided in the Source Data file: Source Data Supplementary Figure1.xlsx.

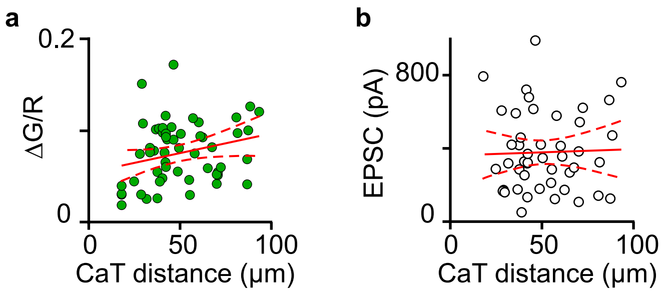

Supplementary Figure 2. Location does not correlate with CaT or EPSC amplitude.

(a) Lack of correlation between CaT amplitude and distance of the CaT from the PCL (n = 62, p = 0.06, $R^2 = 0.06$, simple linear regression). **(b)** Similar plot showing lack of correlation between CF EPSC and distance of the CaT from the PCL (p = 0.84, $R^2 < 0.001$, simple linear regression). Source data are provided in the Source Data file: Source Data Supplementary Figure2.xlsx.

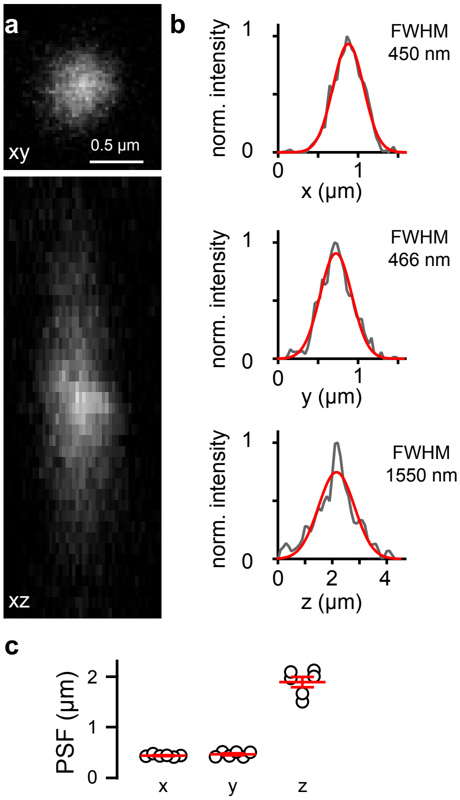

Supplementary Figure 3. Point spread function of 2P microscope.

(a) Average lateral (top) and axial (bottom) images showing the fluorescence of 0.1 µm beads (Tetraspeck™ Microspheres) excited using an 810 nm laser. **(b)** Normalized fluorescence profiles from the images in (a) fit with Gaussian functions. **(c)** Plot of point spread function values measured from individual beads (n = 6). Data are shown as mean ± SEM. Source data are provided in the Source Data file: Source Data Supplementary Figure3.xlsx.

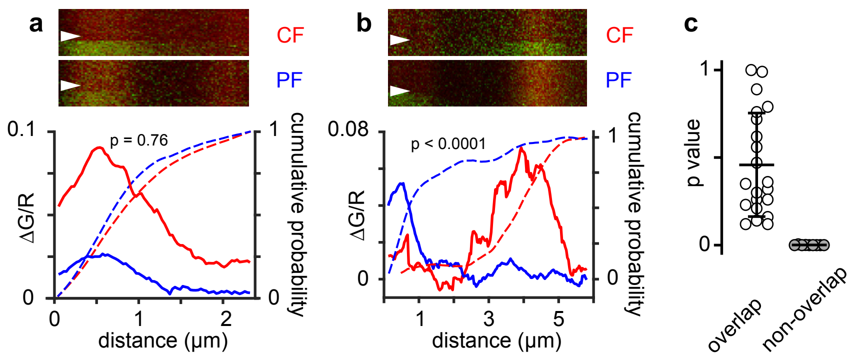

Supplementary Figure 4. Determining overlap using a Two Sample Kolmogorov-Smirnov Test.

(a) Line scans from CF (top, indicated by arrowheads) and PF CaTs (bottom). The fluorescence profile at the peak (solid red and blue lines; range of 3-9 ms post stimulus; 5 ms post stimulus in (a) was normalized and its cumulative probability (dashed lines) was compared using a two Sample Kolmogorov-Smirnov (KS) Test. Sites were overlapping if $p > 0.05$ as in (a) ($p = 0.76$). **(b)** Similar images and analysis for non-overlapping CF and PF CaTs ($p < 0.0001$). **(c)** Two Sample KS Test p values for overlapping ($p > 0.05$: 0.46 ± 0.064, $n = 21$) and non-overlapping ($p < 0.05$: 0.00067 ± 0.00055, $n = 9$) CF and PF CaTs. Data are shown as mean ± SD. Source data are provided in the Source Data file: Source Data Supplementary Figure4.xlsx.

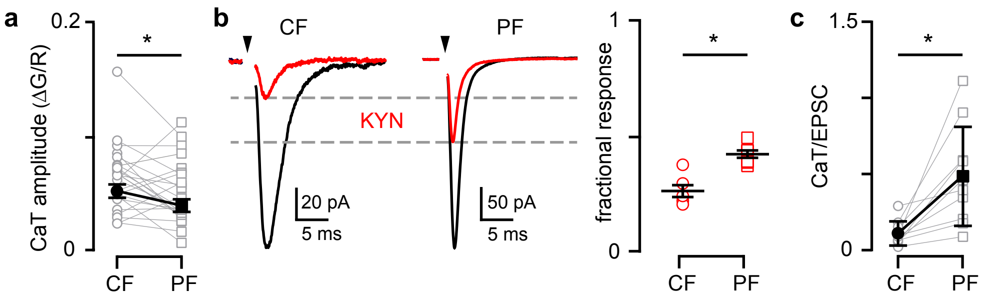

Supplementary Figure 5. [Glutamate] mediating CF and PF CaTs.

(a) CF CaTs were slightly larger than PF CaTs recruited at the same site (0.06 ± 0.006 vs. 0.05 ± 0.006 ΔG/R, n = 24 sites from 20 cells, p = 0.04; Two-tailed paired t test). **(b)** CF and PF EPSCs before (black) and after (red) application of kynurenic acid (KYN; 500 µM). KYN inhibited CF EPSCs more than PF EPSCs (0.26 ± 0.026 vs 0.42 ± 0.016 of control, n = 6, 7, p < 0.001; Two-tailed unpaired t test). **(c)** At overlapping CF/PF sites, the ratio of the CaT amplitude and the charge of the corresponding EPSC integral was lower for CFs than PFs (0.11 ± 0.025 vs. 0.48 ± 0.10 [ΔG/R]/pC, n = 10, p = 0.004; Two-tailed paired t-test). Data are shown as mean ± SEM. Source data are provided in the Source Data file: Source Data Supplementary Figure5.xlsx.

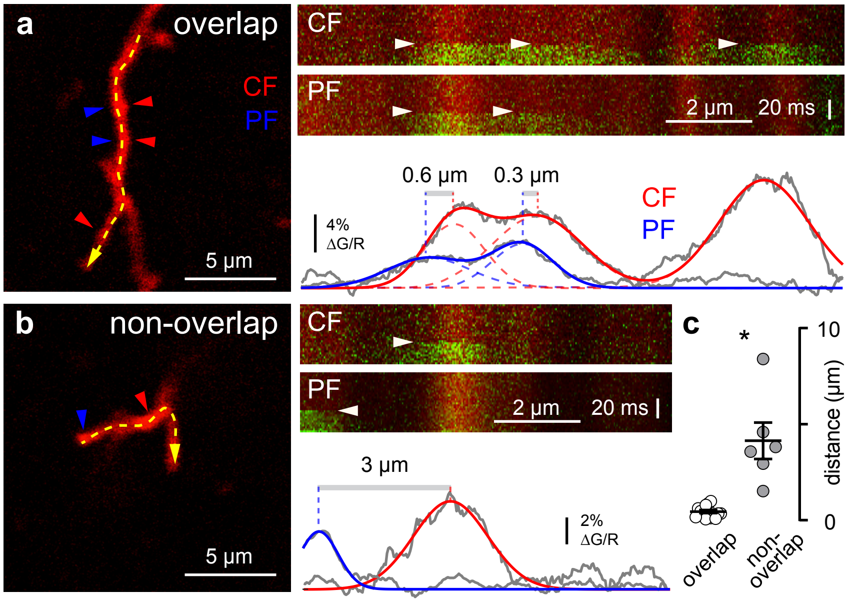

Supplementary Figure 6. Overlapping and non-overlapping CF and PF sites from occlusion experiments.

(a) Dendrite with overlapping CF (red arrowheads) and PF (blue arrowheads) CaTs. White arrowheads on the line scans (top right) indicate discrete CF- or PF-evoked CaTs. Fluorescence profiles (8 ms post stimulus) from each pathway are shown with Gaussian fits overlaid (bottom right) and the distance between overlapping peaks indicated. **(b)** Similar analysis from a dendrite with non-overlapping CF and PF CaTs (6 ms post stimulus). **(c)** Average distance between overlapping (empty circles; 0.45 ± 0.081 µm, n = 14) and non-overlapping sites (gray circles; 4.1 ± 0.95 µm, n = 6). Overlapping sites were significantly closer to one another than non-overlapping sites ($p = 0.01$, Welch's t-test). Asterisk denotes $p < 0.05$. Data are shown as mean ± SEM. Source data are provided in the Source Data file: Source Data Supplementary Figure6.xlsx.

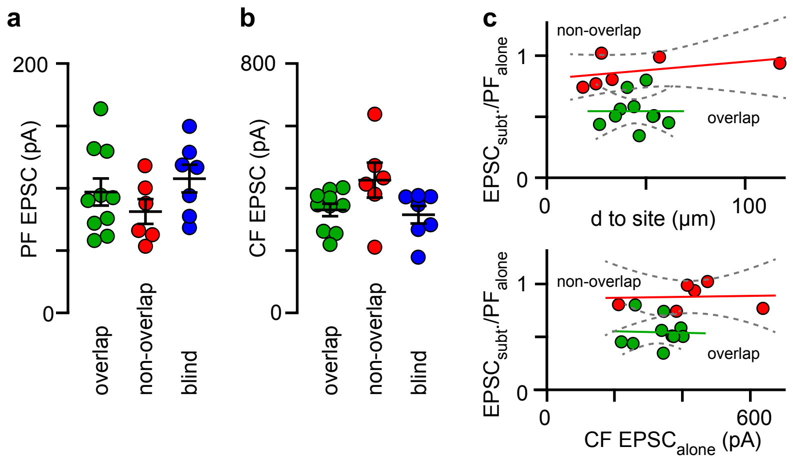

Supplementary Figure 7. Differential voltage control does not affect PF EPSC occlusion.

(a) PF_{alone} EPSC amplitude from overlapping (green; 97.0 ± 10.9 pA), non-overlapping (red; 81.3 ± 9.96 pA), or blindly recruited sites (blue; 108 ± 11.1 pA). **(b)** CF_{alone} EPSC amplitudes were similar across sites (overlap, 331 ± 20.0 pA; non-overlap, 426 ± 56.2 pA; blind, 315 ± 28.0 pA). PF and CF EPSC amplitudes were not significantly different between groups (PF: p = 0.3, CF: p = 0.08, n = 10, 6, and 7, one-way ANOVA). Data are shown as mean ± SEM. **(c, top)** PF EPSC occlusion following CF stimulation (1.3 ms ISI) is not dependent on distance from soma (overlap: green, n = 10, p = 0.9 and non-overlap: red, n = 6, p = 0.4, simple linear regression) or **(c, bottom)** CF EPSC amplitude (overlap: green, n = 10, p = 0.9; non-overlap: red, n = 6, p = 0.9, simple linear regression). Source data are provided in the Source Data file: Source Data Supplementary Figure7.xlsx.
