# Supplementary material for: Afferent convergence to a shared population of interneuron AMPA receptors
Source: Nat Commun. 2023 May 30;14:3113. doi: 10.1038/s41467-023-38854-2 (PMC10229553; doi:10.1038/s41467-023-38854-2)
Supplement: Supplementary file 3 — Reporting Summary [file 41467_2023_38854_MOESM3_ESM.pdf]

nature portfolio

Corresponding author(s): Jacques I. Wadiche

Last updated by author(s): May 3, 2023

Reporting Summary

Nature Portfolio wishes to improve the reproducibility of the work that we publish. This form provides structure for consistency and transparency in reporting. For further information on Nature Portfolio policies, see our Editorial Policies and the Editorial Policy Checklist.

Statistics

For all statistical analyses, confirm that the following items are present in the figure legend, table legend, main text, or Methods section.

n/a	Confirmed	
☐	☒	The exact sample size (*n*) for each experimental group/condition, given as a discrete number and unit of measurement
☐	☒	A statement on whether measurements were taken from distinct samples or whether the same sample was measured repeatedly
☐	☐	The statistical test(s) used AND whether they are one- or two-sided<br>*Only common tests should be described solely by name; describe more complex techniques in the Methods section.*
☒	☐	A description of all covariates tested
☐	☒	A description of any assumptions or corrections, such as tests of normality and adjustment for multiple comparisons
☐	☒	A full description of the statistical parameters including central tendency (e.g. means) or other basic estimates (e.g. regression coefficient) AND variation (e.g. standard deviation) or associated estimates of uncertainty (e.g. confidence intervals)
☐	☐	For null hypothesis testing, the test statistic (e.g. *F*, *t*, *r*) with confidence intervals, effect sizes, degrees of freedom and *P* value noted<br>*Give P values as exact values whenever suitable.*
☒	☐	For Bayesian analysis, information on the choice of priors and Markov chain Monte Carlo settings
☒	☐	For hierarchical and complex designs, identification of the appropriate level for tests and full reporting of outcomes
☒	☐	Estimates of effect sizes (e.g. Cohen's *d*, Pearson's *r*), indicating how they were calculated

Our web collection on statistics for biologists contains articles on many of the points above.

Software and code

Policy information about availability of computer code

Data collection	Electrophysiological data were collected using pClamp 10 software (Molecular Devices). Imaging data were collected using Prairie View software (V5.7; Bruker Corporation).
Data analysis	Electrophysiological and time series Ca2+ imaging data were analyzed using Axograph X software (v1.7 and v1.8). Initial image inspection and processing was performed using ImageJ (v1.53t). Analysis of distances between Ca2+ imaging sites and somas was performed in ImageJ. Custom MATLAB scripts were used to align images, calculate time series data from line scans, and fit line scan images with Gaussian functions. MATLAB scripts are available on github (https://github.com/rpennock/Spill-in-Manuscript-MATLAB). Statistical testing and curve fitting was performed using Graphpad Prism 9.

For manuscripts utilizing custom algorithms or software that are central to the research but not yet described in published literature, software must be made available to editors and reviewers. We strongly encourage code deposition in a community repository (e.g. GitHub). See the Nature Portfolio guidelines for submitting code & software for further information.

Data

Policy information about availability of data

All manuscripts must include a data availability statement. This statement should provide the following information, where applicable:

- Accession codes, unique identifiers, or web links for publicly available datasets
- A description of any restrictions on data availability
- For clinical datasets or third party data, please ensure that the statement adheres to our policy

Source data are included in the manuscript, and raw data are available from the authors upon request.

Human research participants

Policy information about studies involving human research participants and Sex and Gender in Research.

Reporting on sex and gender	n/a
Population characteristics	n/a
Recruitment	n/a
Ethics oversight	n/a

Note that full information on the approval of the study protocol must also be provided in the manuscript.

Field-specific reporting

Please select the one below that is the best fit for your research. If you are not sure, read the appropriate sections before making your selection.

☒ Life sciences ☐ Behavioural & social sciences ☐ Ecological, evolutionary & environmental sciences

For a reference copy of the document with all sections, see nature.com/documents/nr-reporting-summary-flat.pdf

Life sciences study design

All studies must disclose on these points even when the disclosure is negative.

Sample size	For comparisons of physiological and pharmacological properties sample sizes were selected based on what is common in this field of study. Effects were large and consistent between recordings, making large sample sizes unnecessary. The sample size for the comparison of the width of Ca2+ signals along dendrites is consistent with previous work. (Goldberg et al., 2003 Neuron; Soler-Llavina and Sabatini, 2006 Nat Neuro).
Data exclusions	1) Experiments were discarded if series resistance (Rs) of the electrophysiological changed by >20% over the course of the experiment.<br><br>2) If post hoc analysis of evoked spillover (climbing fiber pathway) EPSCs revealed contamination of the signal by parallel fiber activation the recording was discarded.<br><br>3)During imaging of evoked synaptic (parallel fiber pathway) Ca2+ transients experiments were excluded if the location of the Ca2+ transient along the length of the dendrite changed. Such a change indicates that the original synaptic site is no longer being recruited and previously acquired baseline data is invalid.
Replication	Replicates of each experiment are indicated as the sample size 'n'. In cases where multiple data points could be obtained from a single experiment, the number of replicates is indicated by the number of cells recorded from (e.g. n=15 from 10 cells).
Randomization	A mixture of male and female wild-type C57BL/6 mice aged P27-P38 were used in all experiments. Each experimental group contained a random mix of animals of both sexes within the indicated age range.
Blinding	Blinding was not possible for these experiments as all experimental results can be observed in real time and experiments were generally performed and analyzed by the same investigator.

Reporting for specific materials, systems and methods

We require information from authors about some types of materials, experimental systems and methods used in many studies. Here, indicate whether each material, system or method listed is relevant to your study. If you are not sure if a list item applies to your research, read the appropriate section before selecting a response.

Materials & experimental systems

n/a	Involved in the study
☒	☐ Antibodies
☒	☐ Eukaryotic cell lines
☒	☐ Palaeontology and archaeology
☐	☒ Animals and other organisms
☒	☐ Clinical data
☒	☐ Dual use research of concern

Methods

n/a	Involved in the study
☒	☐ ChIP-seq
☒	☐ Flow cytometry
☒	☐ MRI-based neuroimaging

Animals and other research organisms

Policy information about studies involving animals; ARRIVE guidelines recommended for reporting animal research, and Sex and Gender in Research

Laboratory animals	Male and female C57BL/6 mice, aged P27-P38
Wild animals	No wild animals were used in this study.
Reporting on sex	A mixture of male and female mice were used in this study, and sex differences were not considered during design of the experiments.
Field-collected samples	No field collected samples were used in this study.
Ethics oversight	All experimental preparations were performed using protocols approved by the Institutional Animals Care and Use Committee of the University of Alabama at Birmingham.

Note that full information on the approval of the study protocol must also be provided in the manuscript.
